# The Role of Sex and Other Personal Characteristics in the Effects of Symptoms Severity on Self‐Care Agency in Individuals with Multiple Sclerosis

**DOI:** 10.1002/brb3.70091

**Published:** 2024-10-14

**Authors:** Pınar Yeşil Demirci, Saliha Bozdoğan Yeşilot, Zehra Eskimez

**Affiliations:** ^1^ Nursing Department, Faculty of Health Sciences Cukurova University Adana Turkey

**Keywords:** fatigue, multiple sclerosis, self‐care agency, sex, symptom severity

## Abstract

**Background:**

Multiple sclerosis (MS) is a chronic, autoimmune disease that attacks the central nervous system.

**Aims:**

The study was conducted to determine the role of sex and other personal characteristics in the impact of symptom severity on self‐care in individuals with MS.

**Methods:**

The study was cross‐sectional and was completed with 200 participants throughout Turkey. The data were collected through random and snowball sampling using the MS‐related symptom checklist (MS‐RS), the Fatigue Severity Scale (FSS), and the Exercise of Self‐Care Agency (ESCA) form. The data obtained were analyzed using the SPSS 21. Statistical significance was evaluated at the level of *p < *0.05.

**Results:**

The mean age of the participants was 37.97 ± 10.6 years. The mean scores were 47.4 ± 22.41 for MS‐RS, 4.58 ± 2.2 for FSS, and 94.65 ± 24.76 for ESCA in females. The mean MS‐RS score in males was 45 ± 25.89, FSS was 4.33 ± 2.5, and ESCA was 83.43 ± 23.95. There were no significant differences between the sexes except that the ESCA scores were higher in females (*p < *0.05). According to a multiple linear regression analysis, the duration of diagnosis and sensory subdimension of MS‐RS negatively affected the ESCA score in females, and this statistically significant model explained 22.6% of ESCA scores.

**Conclusion:**

The study found that both sexes had clinically significant fatigue, mildly severe symptoms, and moderate self‐care agency. While being a female positively affected self‐care agency, disease duration, and sensory symptoms negatively affected females’ self‐care agency.

## Graphical abstract

This study found that females with multiple sclerosis had better self‐care agency compared to males, but disease duration and sensory symptoms negatively impacted their self‐care. The findings emphasize the need for personalized care strategies considering sex differences in managing multiple sclerosis.

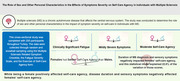

## 1 Introduction

Multiple sclerosis (MS) is a chronic, autoimmune disease that happens when the immune system attacks the brain and spinal cord (World Health Organization 2024). As a severe neurological condition, MS affects around 2.8 million people worldwide (Walton et al. 2020). It is three times more common in women than in men and typically affects young adults between 20 and 30 years old (Gholamzad et al. 2019; Milo and Miller 2014). MS has three clinical subtypes: relapsing‐remitting (RR), secondary progressive (SP), and primary progressive (PP) (Thompson et al. 2018).

MS commonly emerges in the early years through acute relapses followed by partial or complete remission (Gholamzad et al. 2019). It is characterized clinically by discrete episodes of neurologic impairment (Barkhane et al. 2022). The severity of the disease's effects ranges from severe disability within a few years to being free of disability and symptoms for decades or even life (Magyari and Koch‐Henriksen 2022). Sex‐based immunological differences deeply affect MS's onset, course, and prognosis (Angeloni et al. 2021). Men are more subject to the neurodegenerative component of MS than women, particularly after 45 years of age (Magyari and Koch‐Henriksen 2022). Conversely, women suffer more inflammatory disease activity during relapses than men up to menopause, indicating that sex hormones may play a role (Magyari and Koch‐Henriksen 2022; Magyari 2016). Men's and women's differences in immune response regulation are due not only to sex hormones but also to sex‐based genetic differences (Angeloni et al. 2021).

MS symptoms are unpredictable and irregular, can affect any part of the central nervous system, and can cause almost any neurologic symptom (Ghasemi, Razavi, and Nikzad 2017). Furthermore, the symptoms vary widely from person to person and can affect any body part (Ghasemi, Razavi, and Nikzad 2017; NHS 2023). Thus, MS can cause many symptoms, including fatigue, muscle weakness, numbness, and problems with vision, balance, and coordination (Milo and Miller 2014; Barkhane et al. 2022; NHS 2023). One study stated that the symptoms most frequently seen in MS are fatigue (75%–90%), weakness (30.8%), optic neuritis (20.1%), nerve damage (19.6%), and ataxia (14.3%) (Razazian et al. 2020).

Fatigue is one of the most visible and debilitating symptoms of MS, limiting individuals’ social participation and performance at home, at work, and during leisure activities (Khan, Amatya, and Galea 2014; Weiland et al. 2015). MS‐related fatigue is classified as primary or secondary; primary fatigue is linked to specific pathophysiological mechanisms caused by the MS disease process, such as demyelination and axonal loss (Weiland et al. 2015; Gümüş 2018). Secondary fatigue, on the other hand, is associated with sleep disturbances, depression, and side effects of treatment (Gümüş 2018). This research focused on secondary fatigue.

Disease symptom severity can significantly impact the physical disability and cognitive impairment of individuals with MS, making it difficult for them to carry out daily activities and engage in social activities, thereby decreasing their quality of life (Hosseini, Homayuni, and Etemadifar 2022). In addition, with the onset of illness, which might cause a loss of the afflicted individual's job or status, social consequences, such as economic losses, changes in familial relations, and the loss of a spouse through divorce, can affect the afflicted's quality of life as much as their physiological symptoms (Mavioglu et al. 2014). Accordingly, the protection and maintenance of health and improvement of symptoms in individuals with MS are gaining importance. This situation is encapsulated in the concept of self‐care (Mollaoglu, Fertelli, and Tuncay 2006). Dorothea Orem described self‐care as a learned and intentional behavior used to manage development and functioning (Martínez et al. 2021). In addition, everyone can become their own self‐care agent within the restrictions of their condition; this situation manifests as self‐care agency (Martínez et al. 2021). Self‐care agency is an acquired, complex human ability that requires control (Martínez et al. 2021). According to the theory, people practice self‐care to maintain, restore, or improve their health (Khademian, Kazemi Ara, and Gholamzadeh 2020). This theory suggests that self‐care agency should be developed and supported to eliminate the problems and meet the needs of individuals with MS (Mollaoglu, Fertelli, and Tuncay 2006). As a result of the lifestyle changes that develop due to the emergence of MS, self‐care agency may be affected, which may lead to decreased quality of life (Mollaoglu, Fertelli, and Tuncay 2006). As a neurologic condition, MS produces many symptoms that may change during the illness and place a burden on the patient's self‐care. Correlatively, existing symptoms and fatigue levels might affect self‐care agency. However, encouraging a high level of self‐care agency might enhance individuals’ potential to manage their daily lives. Along with symptoms and fatigue, sex might be an important factor that affects the self‐care agency.

This study aimed to determine the role of sex and other personal characteristics in the effects of symptom severity on self‐care agency in individuals with MS.

## 2 Methods

### 2.1 Study Design and Sampling

A cross‐sectional study was conducted with MS participants throughout Turkey. Data were collected between December 1, 2022, and April 30, 2023. The sample for the study was determined using the G*power 3.1.9.7 analysis program (Faul et al. 2007). A priori multiple regression analysis with an effect size of 0.15 (moderate), 22 predictive parameters with 80% power, and a 5% Type 1 error was performed, and the minimum sample size of the study was calculated as 163 participants. The study was completed with 200 participants. The following were the inclusion criteria for participants:

1. Diagnosed with MS for at least 3 months (self‐report with what they see from electronic health system records).
2. Agreed to participate in the study.
3. Aged 18 or over.
4. Literate, with no communication problems.

### 2.2 Data Tools and Data Collection

The data were collected by random and snowball sampling using the Personal Information Form (PIF), the MS‐related symptom checklist (MS‐RS), the Fatigue Severity Scale (FSS), and the Exercise of Self‐Care Agency (ESCA). The questionnaire was created on a web‐based platform using Google Forms. Links to the forms were delivered to participants with MS across Turkey using major social media networks (such as WhatsApp and Facebook) so that they could participate in the study. The researchers asked the participants to share the links with other participants with MS. The transition to the next question without answering the previous question was blocked in the form. Thus, an attempt was made to ensure correct data entry in the form. Each participant's questionnaire completion was predicted to take 7 min: 2 min for the MG‐RS, 2 min for the FSS, and 3 min for the ESCA.

#### 2.2.1 Personal Information Form

A PIF was created on the bais of the relevant literature (Angeloni et al. 2021; Anens et al. 2014; Kister et al. 2021). It consisted of a total of 18 questions: 11 about individuals’ characteristics (age, sex, living place, height, weight, marital status, family type, working status, educational status, income status, and having a child) and seven about specific MS features (type of MS, duration, frequency of attacks, last attack time, additional chronic disease, and medical treatment).

#### 2.2.2 Multiple Sclerosis‐Related Symptom Checklist

The MS‐RS was developed by Gulick et al. (1989) to monitor the symptoms experienced by individuals with MS. A Turkish validity and reliability study of the scale was performed by Tülek et al. in 2017 (Cronbach's alpha = 0.89) (Tülek et al. 2017). The MS‐RS consists of 26 items, with 5 subdimensions to evaluate fatigue—brain stem (4 items), sensory (4 items), neuropsychiatric problems (3 items), excretory problems (6 items), and sleep difficulties (2 items)—and 2 independent (7 items). Scores are assigned between 0 and 130 points on a six‐point scale, and higher points indicate an increased burden of MS‐RSs. In this study, Cronbach's alpha value was 0.939.

#### 2.2.3 Fatigue Severity Scale

The FSS was developed by Krupp et al. (1989), and a Turkish adaptation study was carried out by Armutlu et al. (2007) (Cronbach's alpha = 0.94). The FSS consists of nine questions, with each item scored from 1 to 7. The total score is calculated as the arithmetic mean. A value of 4 or higher indicates severe fatigue. In this study, Cronbach's alpha value was 0.975.

#### 2.2.4 Exercise of Self‐Care Agency

The ESCA scale was developed by American scholars Kearney and Fleischer (1979) based on Orem's self‐care theory. A Turkish validity and reliability study of the ESCA was conducted by Nahcivan in 1993 with healthy young people (Cronbach's alpha = 0.92) (Nahcivan 2004). The ESCA was developed to measure individuals’ self‐care agency. The highest score that can be obtained on the ESCA is 140. The evaluation classifies 24–64 points as bad, 65–100 points as moderate, 101–112 points as good, and 113–140 points as a very good self‐care agency level. In this study, Cronbach's alpha value was 0.926.

### 2.3 Ethical Considerations

Ethical approval (2022/127‐46) was provided by Cukurova University's Scientific Research and Publication Ethics Committee. On the first page of the online survey, the participants were requested to read an informed consent form. Informed consent was gathered from all participants through an “Accept” button on this form. If the participants consented to the survey, they completed the form and selected “Next Page.” They submitted the form after filling it out with the “Submit” button. Because of the form's structure, there was no data loss as the page advanced, and each question was answered.

### 2.4 Data Analysis

The normality of continuous variables was evaluated with the Shapiro–Wilk test. Parametric methods were used for the variables conforming to a normal distribution, and nonparametric methods were used for those that did not. An independent samples *t*‐test and a Mann–Whitney *U*‐test were used to compare two independent groups. The relationships between scores were examined using the Spearman–Rho correlation coefficient. Univariate models were created to evaluate the effects on self‐care capacity one by one, and multiple linear regression models were created to evaluate them together. In addition, multiple linear regression models that included descriptive features were created, and meaningful regression models were obtained using the backward elimination method. Descriptive statistics for continuous variables were expressed as means, standard deviations, medians, quartiles, minimums, and maximums, whereas categorical data were expressed as frequencies and percentages. IBM SPSS 21 was used for the analysis of the data. The statistical significance level was set at 0.05.

## 3 Results

The study was completed with 200 participants: 69.5% of the participants was women, 30.5% was men, and the mean age of the participants was 37.97 ± 10.6. The participants’ mean disease duration was 8.2 ± 6.8 years; 56% had Type 1 MS, 38% had MS attacks rarely, 52% had their last MS attack more than 1 year prior, and 30% had experienced attacks that lasted 1 week (Table 1).

**TABLE 1:** Distribution of sociodemographic characteristics of the participants (*n* = 200).

		Mean ± SD	Min–Max
Age		37.9 ± 10.6	18.0–70.0
BMI		24.7 ± 4.34	15.2–39.0
Disease duration (years)		8.20 ± 6.80	1.00–27.0
		*n*	%
Sex	Female	139	69.5
	Male	61	30.5
Education level	Primary school	21	10.5
	High school	66	33.0
	University	113	56.5
Marital status	Single	66	33.0
	Married	134	67.0
Having children	Yes	127	63.5
	No	73	36.5
Family type	Nucleus	174	87.0
	Extended	26	13.0
Employment status	Non employed	105	52.5
	Employed	95	47.5
Income status	Income less than expenses	65	32.5
	Income equal to expenses	111	55.5
	Income more than expenses	24	12.0
MS type	Relapsing‐remitting	112	56.0
	Secondary progressive	22	11.0
	Primary progressive	18	9.0
	MS type unknown to participants	48	24.0
Attack frequency expression of MS patients	No attack	46	23.0
	Rarely	76	38.0
	Sometimes	64	32.0
	Often	14	7.00
Last MS attack	About a week ago	8	4.00
	About a month ago	21	10.5
	About 6 months ago	32	16.0
	About a year ago	35	17.5
	More than 1 year	104	52.0
Last MS attack duration	A few days	59	29.5
	1 week	60	30.0
	1 month	44	22.0
	More than 1 month	37	18.5
Having additional chronic disease	Yes	68	34.0
	No	132	66.0
Having medical treatment	Yes	167	83.5
	No	33	16.5

Abbreviation: MS, Multiple sclerosis.

The females’ mean total MS‐RS score was 47.4 ± 22.4, total FSS score was 4.58 ± 2.2, and total ESCA score was 94.65 ± 24.76. The males’ mean total MS‐RS score was 45 ± 25.89, FSS score was 4.33 ± 2.5, and ESCA score was 83.43 ± 23.95. There were no significant differences in MS‐RS subdimensions, total scores, and FSS scores by sex, but ESCA scores were higher in females than males (*p < *0.05) (Table 2).

**TABLE 2:** Distribution of multiple sclerosis‐related symptom (MS‐RS), Fatigue Severity Scale (FSS), and Exercise of Self‐Care Agency (ESCA) scores by sex of participants.

	Female			Male			
	Mean ± SD	Median (IQR)	Min–Max	Mean ± SD	Median (IQR)	Min–Max	*p*
Motor	14.7 ± 6.92	15.0 [9.00–19.0]	0–31	14.87 ± 9.19	15.0 [8.00–20.5]	0–35	0.897^a^
Brainstem	7.07 ± 4.51	6.00 [4.00–9.00]	0–20	6.00 ± 4.70	5.00 [2.00–8.50]	0–20	0.081
Sensory	7.36 ± 4.44	7.00 [4.00–12.00]	0–15	6.34 ± 5.01	6.00 [2.00–10.0]	0–15	0.115
Neuropsychiatric	8.94 ± 5.29	8.00 [5.00–13.0]	0–20	8.33 ± 5.43	7.00 [4.00–13.0]	0–20	0.398
Elimination	9.33 ± 6.94	8.00 [4.00–14.0]	0–29	9.46 ± 7.04	9.00 [3.50–14.5]	0–25	0.839
MS‐RS total	47.4 ± 22.41	48.0 [30.0–66.0]	6–98	45.0 ± 25.8	43.0 [26.5–63.0]	0–101	0.371
FSS	4.58 ± 2.20	5.00 [2.89–6.78]	0–7	4.33 ± 2.50	4.22 [2.06–7.00]	0–7	0.877
ESCA	94.6 ± 24.7	98.0 [80.0–113]	25–139	83.4 ± 23.9	88.0 [67.0–100]	31–127	**0.002**

*Note*: *p*: Mann–Whitney *U*‐test. Bold element is used in the meaning of statistically significant.

^a^Inpedependent Samples *t*‐test.

A multiple linear regression model was established to evaluate the effects of age, body mass index, educational level, marital status, having children, family type, employment status, income level, disease duration, attack frequency, MS type, last attack time, duration of last attack, presence of another chronic disease, previous medical treatment, MS‐RS status (motor, brainstem, sensory, neuropsychiatric, or elimination problems), and FSS status on ESCA scores using the backward elimination method. The model was significant (*p < *0.05). According to the regression analysis, sex was a significant predictor, and being female positively affected ESCA values. In females, being a primary and high school graduate, disease duration, having had an MS attack within the last 6 months, and having had MS attack durations of a few days affected ESCA scores negatively, whereas being married had a positive effect. The variables in the model explained 41.7% of the ESCA values (*p < *0.001). In males, being a primary and high school graduate, having children, being employed, not having had an MS attack, having MS Type 2, having had the last MS attack within the previous 13 months, having received medical treatment, and having an MS‐RS sensory subdimension affected the ESCA values negatively, whereas being married and having MS Type 3 had positive effects. Having had an MS attack within the previous week, attack durations of a few minutes/hours, and durations of a few days were not detected on ESCA (*p* > 0.05). The variables in the model explained 62.2% of the ESCA (*p < *0.001) (Table 3).

**TABLE 3:** Determinants of Exercise of Self‐Care Agency (ESCA) according to multiple linear regression analysis.

					95.0% confidence interval for B		
		Unstandardized coefficients (B)		Standardized coefficients (beta)	Lower bound	Upper bound	*p*
All group *R* ^2^: 0.348 *F*: 7.649 ***p* < 0.001**	(Constant)	86.6	7.94		71.0	102	< 0.001
	Age	0.45	0.20	0.19	0.07	0.84	**0.022**
	Sex (female)	7.83	3.45	0.15	1.02	14.6	**0.025**
	Primary school	−14.9	5.22	−0.18	−25.2	−4.65	**0.005**
	High school	−7.71	3.48	−0.15	−14.6	−0.86	**0.028**
	Marital status (married)	15.2	4.29	0.29	6.81	23.7	**< 0.001**
	Having children (yes)	−14.9	4.84	−0.29	−24.5	−5.40	**0.002**
	Disease duration (years)	−0.83	0.26	−0.23	−1.40	−0.31	**0.002**
	Secondary progressive	−12.5	5.31	−0.16	−23.0	−2.10	**0.019**
	Last MS attack (about 6 months ago)	−9.37	4.35	−0.14	−17.9	−0.79	**0.032**
	Last MS attack (about a year ago)	−7.24	4.17	−0.11	−15.5	0.98	0.084
	Attack duration (a week)	10.1	3.42	0.19	3.40	16.9	**0.003**
	Sensory	−1.72	0.38	−0.32	−2.50	−0.97	**< 0.001**
	Elimination	0.57	0.26	0.16	0.06	1.09	**0.028**
Female *R* ^2^: 0.417 *F*: 9.163 *p* < 0.001	(Constant)	116	5.31		105	126	**< 0.001**
	Primary school	−20.4	6.64	−0.22	−33.6	−7.30	**0.003**
	High school	−11.8	3.80	−0.22	−19.4	−4.33	**0.002**
	Marital status (married)	17.3	4.00	0.32	9.41	25.2	**< 0.001**
	Disease duration (years)	−0.95	0.29	−0.24	−1.53	−0.38	**0.001**
	Last MS attack (about 6 months ago)	−13.0	4.47	−0.21	−21.8	−4.12	**0.004**
	Attack duration (a few days)	−15.9	5.04	−0.25	−25.9	−5.94	**0.002**
Male *R* ^2^: 0.622 *F*: 4.930 ***p* < 0.001**	(Constant)	162	12.5		137	187	< 0.001
	Primary school	−26.0	7.43	−0.40	−40.6	−10.6	**0.001**
	High school	−10.9	5.76	−0.22	−22.5	0.73	**0.066**
	Marital status (married)	23.6	7.96	0.49	7.53	39.6	**0.005**
	Having children (yes)	−36.8	8.23	−0.74	−53.4	−20.2	**< 0.001**
	Employed	−25.5	5.81	−0.53	−37.2	−13.8	**< 0.001**
	Having no attack	−19.8	6.29	−0.37	−32.5	−7.10	**0.003**
	Secondary progressive	−26.9	6.65	−0.45	−40.3	−13.5	**< 0.001**
	Primary progressive	28.2	9.64	0.35	8.80	47.6	**0.005**
	Last MS attack (about a week ago)	−27.8	13.9	−0.21	−55.8	0.16	0.051
	Last MS attack (about a month ago)	−34.5	9.10	−0.46	−52.8	−16.1	**< 0.001**
	Last MS attack (about a year ago)	−22.4	6.65	−0.36	−35.8	−9.00	**0.002**
	Attack duration (a few minutes/hours)	−18.5	9.95	−0.25	−38.6	1.52	0.069
	Attack duration (a few days)	−10.3	5.88	−0.19	−22.12	1.51	0.086
	Having medical treatment	−18.2	6.61	−0.31	−31.5	−4.90	**0.008**
	Sensory	−1.60	0.54	−0.32	−2.64	−0.46	**0.006**

*Note*: B:regression coefficient, *R* ^2^: explanatory coefficient. Bold element is used in the meaning of statistically significant.

Abbreviation: MS, multiple sclerosis.

The determination of ESCA by age, MS‐RS value, FSS score, and disease duration are shown in Table 4. In Model 1, the effects of age, sex, MS‐RS subdimensions, and FSS scores on ESCA values were examined individually. Although being a female affected ESCA scores positively, the duration of diagnosis, as well as brainstem, sensory, and fatigue FSS scores ≥ 4, affected them negatively (*p < *0.05). In Model 2, the interactions between these variables were taken into account, and the effect on ESCA scores was reexamined. Accordingly, although being a female positively affected ESCA values, the duration of diagnosis and sensory subdimension of MS‐RS had a negative effect (*p < *0.05). Although the negative effects of the brainstem and FSS scores ≥ 4 persisted, their significance in the model disappeared. The new model explained 18.2% of the ESCA score (Table 4).

**TABLE 4:** Determinants of Exercise of Self‐Care Agency (ESCA) by sex, multiple sclerosis‐related symptom (MS‐RS), Fatigue Severity Scale (FSS), and disease duration in participants.

	Model 1: Univariate					Model 2: Multiple				
			95.0% confidence interval for B					95.0% confidence interval for B		
	Unstandardized coefficients (B)	Standardized beta coefficients (Beta)	Lower bound	Upper bound	*p*	Unstandardized coefficients (B)	Standardized beta coefficients (beta)	Lower bound	Upper bound	*p*
Age	0.18	0.08	−0.15	0.51	0.274	0.17	0.07	−0.19	0.53	0.354
Sex	11.2	0.21	3.80	18.7	**0.003**	12.2	0.23	4.95	19.5	**0.001**
Motor	−0.40	−0.12	−0.86	0.05	0.081	0.37	0.11	−0.29	1.03	0.271
Brainstem	−1.02	−0.19	−1.77	−0.27	**0.008**	−0.64	−0.12	−1.61	0.33	0.197
Sensory	−1.54	−0.29	−2.27	−0.82	**< 0.001**	−1.65	−0.31	−2.63	−0.67	**0.001**
Neuropsychiatric	−0.63	−0.13	−1.28	0.02	0.057	0.14	0.03	−0.79	1.06	0.772
Elimination	−0.31	−0.09	−0.81	0.19	0.224	0.15	0.04	−0.49	0.80	0.640
FSS ≥ 4	−8.51	−0.17	−15.56	−1.46	**0.018**	−3.60	−0.07	−11.5	4.30	0.370
Disease duration	−0.61	−0.17	−1.12	−0.10	**0.019**	−0.67	−0.18	−1.20	−0.14	**0.013**
	*R* ^2^ age: 0.006. *R* ^2^ sex: 0.043. *R* ^2^ motor: 0.015. *R* ^2^ brainstem: 0.035. *R* ^2^ sensory: 0.082. *R* ^2^ neuropsychiatric: 0.018. *R* ^2^ elimination: 0.007. *R* ^2^ FSS: 0.028. *R* ^2^ disease duration: 0.027					*R* ^2^: 0.182. *p* < 0.001				

*Note*: B:regression coefficient, *R* ^2^: explanatory coefficient. Bold element is used in the meaning of statistically significant.

The effects of age, duration of diagnosis, MS‐RS scores, and FSS values on ESCA scores in females and males were evaluated together. The duration of diagnosis and sensory subdimension of MS‐RS negatively affected the ESCA score in females, and this statistically significant model explained 22.6% of ESCA scores (*p < *0.001). On the other hand, no parameters affected the ESCA scores in males, and the model created was not statistically significant (*p* = 0.909) (Table 5).

**TABLE 5:** Determinants of Exercise of Self‐Care Agency (ESCA) by sex, multiple sclerosis‐related symptom (MS‐RS), Fatigue Severity Scale (FSS), and disease duration in females and males.

	Female					Male				
			95.0% confidence interval for B					95.0% confidence interval for B		
	Unstandardized coefficients (B)	Standardized beta coefficients (beta)	Lower bound	Upper bound	*p*	Unstandardized coefficients (B)	Standardized beta coefficients (beta)	Lower bound	Upper bound	*p*
(Constant)	87.1		56.2	118	< 0.001	105		87.6	123	< 0.001
Age	0.14	0.06	−0.62	0.90	0.709	0.22	0.09	−0.21	0.65	0.319
Motor	−0.14	−0.05	−1.34	1.07	0.821	0.66	0.18	−0.16	1.48	0.115
Brainstem	−0.10	−0.02	−2.09	1.88	0.918	−0.69	−0.13	−1.85	0.48	0.245
Sensory	−0.88	−0.18	−2.67	0.91	0.328	−2.10	−0.38	−3.33	−0.87	**0.001**
Neuropsychiatric	0.60	0.14	−1.47	2.67	0.564	−0.33	−0.07	−1.42	0.75	0.545
Elimination	−0.37	−0.11	−1.81	1.07	0.609	0.40	0.11	−0.33	1.14	0.280
FSS ≥ 4	3.23	0.07	−13.6	20.0	0.702	−5.01	−0.10	−14.3	4.29	0.289
Disease duration	−0.43	−0.13	−1.38	0.52	0.367	−0.78	−0.20	−1.46	−0.10	**0.025**
	*R* ^2^: 0.226. *p* < 0.001					*R* ^2^: 0.060. *p* = 0.909				

*Note*: *p*: multiple linear regression. B: regression coefficient, *R* ^2^: explanatory coefficient. Bold element is used in the meaning of statistically significant.

## 4 Discussion

This study examined the role of sex and relevant patient characteristics in the effects of symptom severity on self‐care agency in individuals with MS. It was determined that both sexes had clinically significant fatigue, mild symptom severity, and moderate self‐care agency.

The study showed that the female and male participants had similar fatigue levels. Kister et al. (2021), in a study of MS symptoms by age, sex, and race/ethnicity, determined that women had greater fatigue than men. Elsewhere, fatigue was reported as a significant issue for both sexes, which is consistent with this study's results (Anens et al. 2014; Moore et al. 2022).

The present study found that females and males had mild symptom severity. Kister et al. (2021) reported that symptom severity was similar in both sexes of individuals with MS. In a study of symptom severity in individuals with MS, of which 70.9% were women, Lallana et al. (2019) reported that the individuals’ symptoms were mild. As in the related literature, the mild symptom severity of the males and females in the present study may have been due to the low average age of the participants (Kister et al. 2021; Lallana et al. 2019).

In the present study, both sexes had moderate self‐care agency, but the females’ self‐care agency was significantly higher. Mollaoglu, Fertelli, and Tuncay (2006), in a study of self‐care agency and the factors that affect individuals with MS, determined that the self‐care agency of individuals with MS was insufficient. Bayram and Yurttaş (2022) determined that self‐care agency levels in individuals with MS were higher than moderate. In the literature, self‐care is defined as a learned and intentional behavior used to manage development and functioning, and everyone can become their self‐care agent within the restrictions of their condition (Martínez et al. 2021). Individuals with MS need to find a way to live and cope with the condition healthily through effective self‐care. The literature indicates that numerous factors influence an individual's self‐care agency or ability to care for themselves, including age, sex, sociocultural standing, family structure, lifestyle, and resource access (Karadağ, Caliskan, and Baykara 2017). It is unknown whether females and males have different needs regarding care, therapy, and support due to sex and the different stages of the disease (Schermann et al. 2023). Therefore, the factors affecting self‐care may also vary in individuals with MS. In women in the current study, self‐care agency was affected negatively by being a primary and high school graduate, disease duration, having an MS attack within the last 6 months, and having an MS attack duration of a few days. In men, self‐care agency was affected negatively by being a primary and high school graduate, having children, being employed, not having an MS attack, having MS Type 2, having had the last MS attack within the last 13 months or so, having received medical treatment, and exhibiting the MS‐RS sensory subdimension. These factors were found to negatively affect the self‐care agency of women (41.7%) and men (62.2%); the degrees to which these factors explained self‐care agency differed by sex. The fact that these disease‐related factors negatively affect the self‐care agency of individuals with MS was an expected result, and the regression analysis also supported these results. It was also determined that being married positively affected self‐care agency in both sexes. Although they studied different disease groups, Asadi et al. (2019) found a relationship between marital status and self‐care ability, with higher scores in unmarried people. In the current study, the reason that being married had a positive effect on self‐care agency in both sexes may have been that married couples share family responsibilities, which could enhance self‐care abilities.

In this study, MS‐RS, FSS, and disease duration were analyzed as determinants of self‐care agency. Model 1 showed that, although being female affected self‐care agency positively, the duration of diagnosis as well as brainstem, sensory, and fatigue FSS scores ≥ 4 affected it negatively. Furthermore, when the interactions between these variables and their effects on self‐care agency were examined, being female positively affected self‐care agency, whereas the duration of diagnosis and sensory subdimension of MS‐RS had a negative effect. Although the negative effect of the brainstem and FSS scores ≥ 4 continued, their significance in the model disappeared. The new model was used to calculate that these factors explained 18.2% of self‐care agency.

In Turkish society, women grow up seeing that all the other women around them are caring. Their mothers and grandmothers meet all the care needs of children, adults, and the home, and these women expect the girls they raise in their own homes to do the same. Traditional gender roles are instilled in girls as they watch older women, adopt them as role models, and try to meet their expectations. Therefore, these expectations start early and can lead women to take responsibility at a young age, developing certain practices and skills because they do certain things constantly. Although somewhat less commonly today, care expectations that correlate with gender roles are explicitly expected after marriage. Even if educated, women may try to meet these expectations. This situation may cause women to feel strong, even if they are sick, as they have developed practical skills: being able to meet more needs with less energy, being good wives and mothers according to their understanding of these roles, and possessing a strong sense of personal competence created by developing these skills.

Additionally, the effects of age, diagnosis duration, MS‐RS scores, and FSS scores on self‐care agency in females and males were evaluated together in the current study. The duration of diagnosis and sensory subdimension of MS‐RS negatively affected self‐care agency in females, and the corresponding, statistically significant model explained 22.6% of self‐care agency. On the other hand, no parameters affected self‐care agency in males. Magyari and Koch‐Henriksen (2022) study of sex differences in individuals with MS found that women exhibited more inflammatory disease activity, with 16% higher recurrence rates than men. Moreover, the difference in inflammatory disease activity between men and women disappeared after age 50. Even these biological differences can affect individuals’ self‐care agency regarding disease progression in the present study. According to the latest International Multiple Sclerosis Federation (MSIF, 2021) data, the number of female individuals with MS is at least twice as high as that of men (Multiple Sclerosis International Federation [MSIF] 2021). Although MSIF (2021) research suggests that women are more affected by the disease than men up to a certain age, this difference in self‐care agency in the present study may also have been affected by the low average age of the individuals.

## 5 Conclusion and Recommendations

According to this study's results, both sexes had clinically significant fatigue, mild symptom severity, and moderate self‐care agency, but females’ self‐care agency was significantly higher than that of males. Disease duration and the presence of sensory symptoms had a negative effect on females' self‐care agency. On the bais of these results, the following recommendations are proposed. For providing personalized MS care in the healthcare system, researchers should be carried out more individuals with MS to reveal the sex difference. Nurses should be aware that the capabilities of individuals with MS self‐care agency might affect how they should be cared for. Nurses should develop specific approaches to enhance self‐care agency in individuals with MS. Studies are needed to evaluate the effectiveness of different methods of increasing quality of life and self‐care skills in individuals with MS.

## 6 Limitations

The results cannot be generalized to patient groups currently receiving treatment in the patient clinic. Another limitation of this study may be that the data were collected online, and 200 participants used the digital domain.

## Author Contributions

**Pınar Yeşil Demirci**: conceptualization, data curation, methodology, formal analysis, writing–original draft. **Saliha Bozdoğan Yeşilot**: conceptualization, methodology, data curation, writing–review & editing, formal analysis. **Zehra Eskimez**: conceptualization, methodology, data curation, writing–review & editing.

## Conflicts of Interest

The authors declare no conflicts of interest.

### Peer Review

The peer review history for this article is available at https://publons.com/publon/10.1002/brb3.70091.

## Data Availability

The data that support the findings of this study are available from the corresponding author upon reasonable request.
